# Investigating public behavior with artificial intelligence-assisted detection of face mask wearing during the COVID-19 pandemic

**DOI:** 10.1371/journal.pone.0281841

**Published:** 2023-04-11

**Authors:** Kasem Seresirikachorn, Paisan Ruamviboonsuk, Ngamphol Soonthornworasiri, Panisa Singhanetr, Titipakorn Prakayaphun, Natsuda Kaothanthong, Surapoom Somwangthanaroj, Thanaruk Theeramunkong

**Affiliations:** 1 Sirindhorn International Institute of Technology, Thammasat University, Pathumthani, Thailand; 2 Department of Ophthalmology, College of Medicine, Rajavithi Hospital, Rangsit University, Bangkok, Thailand; 3 Faculty of Tropical Medicine, Department of Tropical Hygiene, Mahidol University, Bangkok, Thailand; 4 Department of Ophthalmology, Mettapracharak Hospital, Nakhon Pathom, Thailand; 5 Department of Constructional Engineering, Graduate School of Engineering, Chubu University, Kasugai, Japan; 6 The Royal Society of Thailand, Bangkok, Thailand; Hanyang University, REPUBLIC OF KOREA

## Abstract

**Objectives:**

Face masks are low-cost, but effective in preventing transmission of COVID-19. To visualize public’s practice of protection during the outbreak, we reported the rate of face mask wearing using artificial intelligence-assisted face mask detector, AiMASK.

**Methods:**

After validation, AiMASK collected data from 32 districts in Bangkok. We analyzed the association between factors affecting the unprotected group (incorrect or non-mask wearing) using univariate logistic regression analysis.

**Results:**

AiMASK was validated before data collection with accuracy of 97.83% and 91% during internal and external validation, respectively. AiMASK detected a total of 1,124,524 people. The unprotected group consisted of 2.06% of incorrect mask-wearing group and 1.96% of non-mask wearing group. Moderate negative correlation was found between the number of COVID-19 patients and the proportion of unprotected people (r = -0.507, *p*<0.001). People were 1.15 times more likely to be unprotected during the holidays and in the evening, than on working days and in the morning (OR = 1.15, 95% CI 1.13–1.17, *p*<0.001).

**Conclusions:**

AiMASK was as effective as human graders in detecting face mask wearing. The prevailing number of COVID-19 infections affected people’s mask-wearing behavior. Higher tendencies towards no protection were found in the evenings, during holidays, and in city centers.

## Background

Coronavirus disease (COVID-19) caused by the infection of coronavirus 2 (SARS-CoV-2), leads to severe acute respiratory syndrome. The first reported cluster was from Wuhan, China, in January 2019 [1,2]. As of August 16, 2022, there have been over 590 million confirmed cases of COVID-19, with over 6 million deaths [3]. This coronavirus pandemic is affecting not only the health and well-being of the population, but also socioeconomic aspects [4–7]. COVID-19 is transmitted via droplets from symptomatic and asymptomatic infected people [8–11]. Primary prevention is also an important aspect of disease control; therefore, protection against virus transmission through droplets is vital [12–14].

Wearing a face mask can protect against droplet transmission of many diseases including COVID-19 [15]. There is ample evidence to show that face masks can reduce infection rates and mortality rates at a cost-effective level [16–18], and current WHO guidelines recommend wearing them [19]. Studies have proved that mask wearing reduces the spread of the virus, especially when compliance is high [20,21]. Previous studies evaluated the number of people wearing face mask through manual counting and self-reported questionnaires, which are subjective and requires continuous manual labor, which is inconvenient and adds the risk of exposure to droplet-borne infections during this time of social distancing [22,23]. Artificial intelligence (AI)-assisted systems can come into play to help visualize the public’s awareness of mask wearing to give a better picture as to whether there is adequate practice of protection during the outbreak.

Bangkok has been reported as the city with the highest number of COVID-19 patients in Thailand [24]. The Department of Public Health, Ministry of Higher Education, Science, Research, and Innovation together with Sirindhorn International Institute of Technology, Thammasat University, have developed an AI-assisted face mask detector, and it has been used since January 23, 2021.

This research uses Ai-assisted face mask detector to see whether the number of mask wearers correlate with the number of newly reported COVID-19 cases in Bangkok.

## Materials and methods

The study protocol was approved by the institutional review boards of Rajavithi Hospital, Thailand and of Sirindhorn International Institute of Technology (SIIT), Thammasat University. Patient’s informed consent was exempted by the IRB and this study was conducted in accordance with the Declaration of Helsinki.

### AI-assisted face mask wearing (AiMASK)

**Fig 1:**
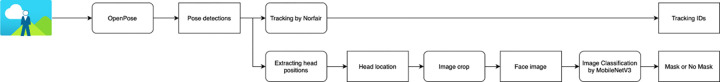
An end-to-end flowchart of the AiMask model.

AiMASK system was developed using OpenPose for pose detection, Norfair for human tracking, and MobileNetV3 for mask detection. The OpenPose is a real-time multi-person system which detects human body, hand, face, and foot key-points (in total 135 key-points) on single images. The Norfair is a Python library for real-time 2D object tracking built by Tryolabs. It predicts each point’s future location based on previous positions and align these approximate locations with the detector’s newly observed points to perform tracking. The MobileNetV3 was used with TensorFlow Lite to detect masks (Fig 1).

Images were categorized into protected and unprotected group. The protected group was the correct mask-wearing group, and the unprotected group consisted of incorrect mask-wearing and non-mask-wearing people. The correct mask-wearing group consisted of people whose face masks covered their mouth, nose, and chin simultaneously, while the incorrect mask-wearing group was composed of people who wore a face mask that did not cover their mouth, nose, and chin at the same time. The non-mask-wearing group was made up of those whose face mask was not detected (S1 Fig).

All images for training and validation were attained through closed-circuit televisions (CCTVs) files owned by the Bangkok Metropolitan, which we received permission from the Bangkok Metropolitan to access all the CCTV files.

For the last process we utilized 8,551 images obtained from CCTVs around the city during December 2020, which were independent images from the ones used for the training of AiMask. Initially, 6,795 images were used for training. AiMASK marked 3,180 images as protected group and 3,615 images as unprotected. (Table 1) After successful training, internal validation was performed using the testing set of images which comprised of around 20% (1,756 images) of the initial images. During internal validation, average accuracy of AiMASK was 97.83% (95%CI; 97.04–98.46%) (Table 1).

**Table 1:** Overall performance of AiMASK on training and testing datasets.

Mask-wearing group	Total (N = 8,551)	Training set (N = 6,795, 79.46%)	Testing set (N = 1,756, 20.54%)			
			**Sample Images**	**AiMASK**	**Accuracy**	**CI 95%**
**Protected group**	3,960	3,180	780	771	98.85%	97.82–99.47%
**Unprotected group**	4,591	3,615	976	947	97.03%	95.76%-98%

**Fig 2:**
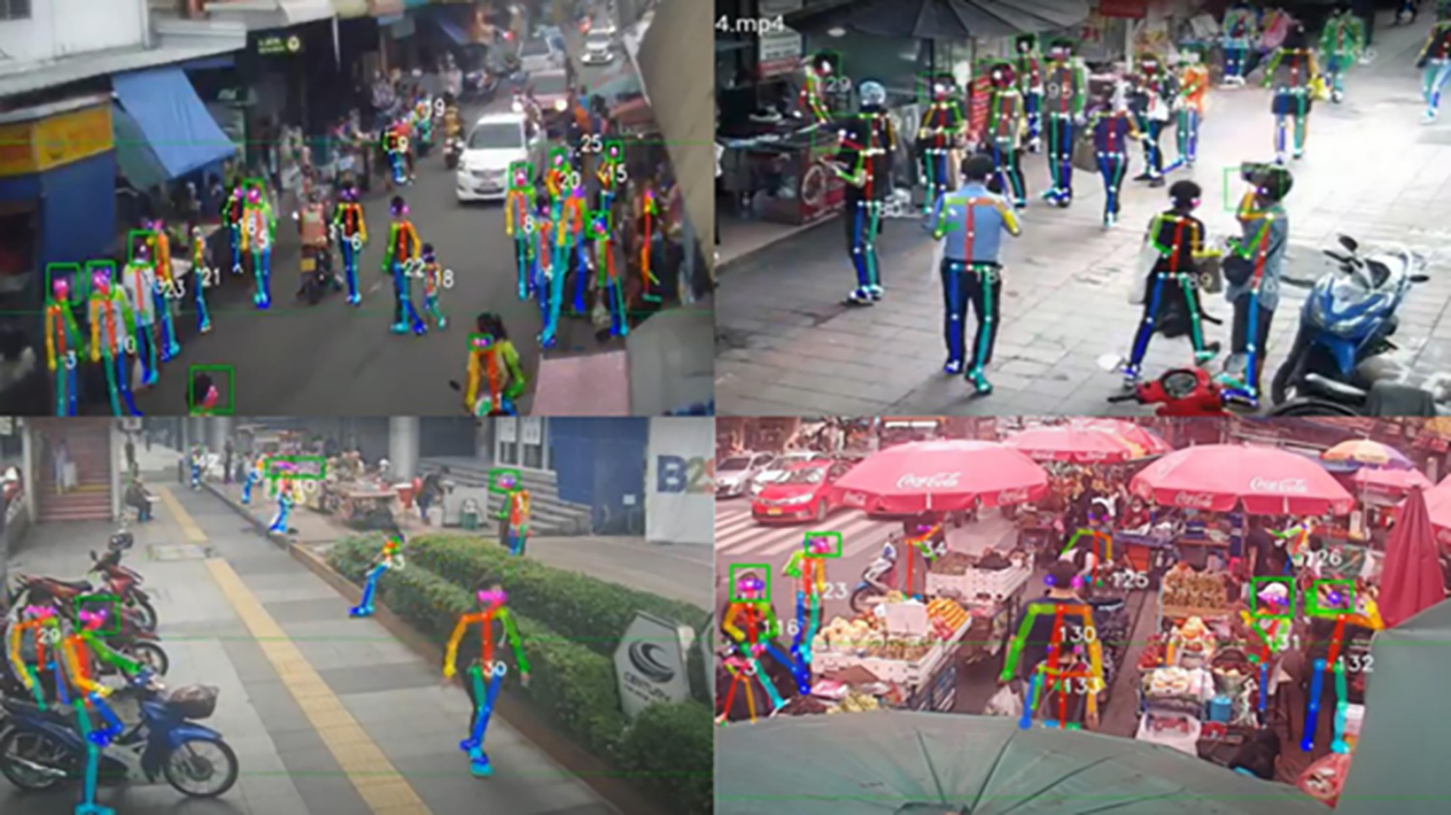
Sample of debugged images for validating results.

After training and internal validation was completed, external validation was done on an independent data set of randomly selected images from 64 (one-hour) CCTV files from the morning and in the evening from each of the 32 districts from different dates and time during 20–22 January 2021 and compared the results of the human graders with those of AiMASK (Fig 2).

### AiMASK data collection

Having been verified internally and externally, AiMASK was used to gather information for this study. Data classified into the unprotected group were manually allocated into the non-mask-wearing and incorrect mask-wearing groups. AiMASK analyzed recorded videos from the same CCTVs the training images were gathered from. Files from CCTVs in various areas in every district around Bangkok were sent to AiMASK for evaluation. Each file collected had a duration of one hour, and all data were updated on the AiMASK website daily (https://aimask.aiat.or.th/).

AiMASK detected images from public areas between January 23, 2021, and April 22, 2021 (90 days). Videos were taken from two separate timeframes, one during the morning (7am-8am), and one during the evening (5pm-6pm). We subcategorized days into working days and holidays, with working days defined as Monday to Friday, and holidays defined as weekends and public holidays. We also subcategorized data by type of place from which images were gathered into 7 categories: market entrances; inside the markets; public transportation (bus stops and sky trains); malls and convenience store entrances; building entrances; footbridges; and along the sidewalk. Districts were subdivided into 2 groups, the city center and suburban districts.

Numbers of COVID-19 patients were taken from the daily official reports from Bangkok Metropolitan Data Center which reveals new cases over a 24-hour period. New clusters of COVID-19 in this research comprised of 2 big events announced by the Department of Disease Control under the Ministry of Public Health. The first cluster was reported on March 14, 2021, from Bang Kae, and the second cluster was in Thonglor on April 5, 2021. Patients who tested positive were reported according to their current residential district.

### Outcome measures

Data were categorized into two groups for analysis: the protected group and the unprotected group. The primary outcome was the proportion of the protected and unprotected groups together with correlations with the reported number of COVID-19 infections. Secondary outcome was identification of factors showing correlations with the varying proportions of the unprotected group.

### Statistical analysis

Descriptive statistics were used to report the total number of people analyzed by AiMASK and the proportion of mask wearing. Continuous data were reported using mean, median, and standard deviation (SD). External validation of AiMASK was analyzed by confusion matrix. The accuracy, precision, recall, and F1 scores were calculated. Correlations were calculated by Pearson’s correlation coefficient. A “very high” correlation was defined as a correlation coefficient of 0.90–1.00, a “high” correlation was a value of 0.70–0.89, a “moderate” correlation was defined as a correlation coefficient of 0.50–0.69, and a “low” correlation was a value of 0.30–0.49. Little or no correlation was considered to be a correlation coefficient ≤ 0.29.

Categorical variables were compared using the Chi-Square test. Data were analyzed using univariate analysis with 95% confidence interval (CI), and *p*<0.05 was considered statistically significant. All analyses were performed with SPSS 16.0 for Windows (SPSS Inc., Chicago, IL, USA).

## Results

### External validation of AiMASK

AiMASK classified 3,000 people into the same group as human graders, giving AiMASK an accuracy of 91%. F1 score was 0.91 in both groups (Table 2).

**Table 2:** External validation of AiMASK (n = 3,000).

N = 3,000		AiMASK			
		Protected group	Unprotected group	Total	Recall
**Human**	**Protected group**	1,391	174	1,565	0.89
	**Unprotected group**	87	1,348	1,435	0.94
	**Total**	1,478	1,522	3,000	
	**Precision**	0.94	0.89		

### Overall data

During the 90 days of the study, 1,124,524 people were counted. The protected group accounted for the largest proportion (95.98%), followed by the unprotected group (4.02%). Incorrect mask wearing and the non-mask-wearing groups constituted 2.06% and 1.96% respectively. The protected group was over 90% at every time point. The average number of places analyzed per day was 24.87 ± 5.34, and the average number of people detected per day was 12,494 ± 3044.63.

During the same 90-day timeframe, the total number of new COVID-19 patients was 6,312. The median number of new daily cases was 15.5 (range 0–446). Two weeks before the Bang Kae cluster, the size of the unprotected group increased to 5.31%. During the same two weeks before the first cluster, an average of 4.2 cases were reported per day. The highest percentage of people in the unprotected group was 8.38% on March 14, 2021, the same day the Bang Kae cluster was announced. A day later, the size of the unprotected group decreased to 4.74%.

**Fig 3:**
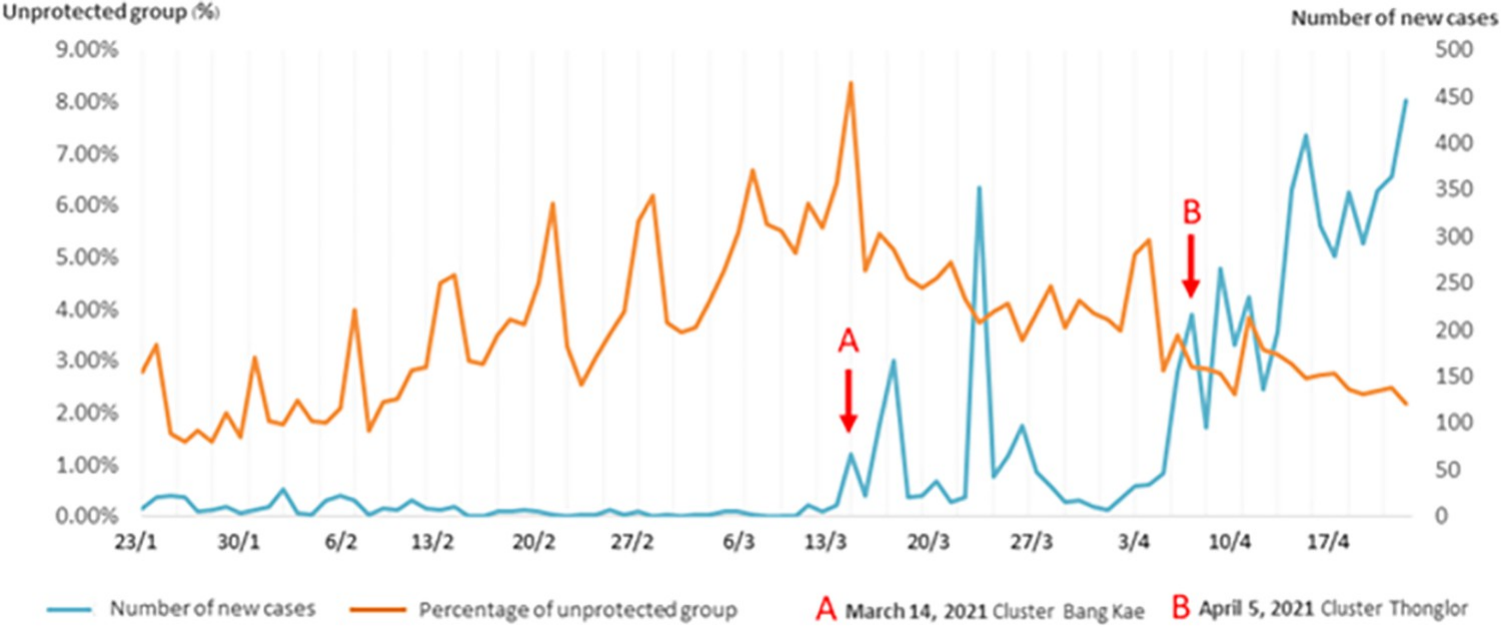
Correlation between the unprotected group and reported new COVID-19 patients.

Twenty-three days after the Bang Kae cluster, another cluster was announced in Thonglor area (April 5, 2021). On that day, the unprotected group accounted for 3.51% of the observed individuals. One day after the Thonglor cluster, the unprotected group decreased to just 2.90%. Ever since this second cluster, the proportion of people in the unprotected group varied between 2.18% and 3.84%. After the Thonglor cluster, the mean number of cases reported per day was 272.9 (Fig 3).

Moderate positive correlation was found between the number of new COVID-19 patients and the number of protected group (r = 0.432, *p*<0.001), while negative correlation was found between the number of new COVID-19 patients and the size of the unprotected group (r = -0.507, *p*<0.001). Overall, there was a moderate negative correlation between the amount of new COVID-19 patients and the proportion of people in the unprotected group.

### Face mask wearing divided by place

The percentage of unprotected individuals in all 7 types of places showed statistically significant differences (*p*<0.001). The lowest percentage of unprotected individuals was found inside markets at 2.64%. The Odds Ratio (OR) of building entrances was 2.30 (95% CI 2.20–2.41, p<0.001), while the OR of the sidewalk was 1.88 (95% CI 1.80–1.96, *p*<0.001) compared with inside markets (Table 3).

**Table 3:** Univariate logistic regression analysis of the unprotected group divided into type of public place, date, time, and districts.

Variables	Total (%) N = 1,124,524	%Unprotected (%Incorrect, %Non-mask)	OR	CI 95%	p-value
**Type of place**					
Building entrances	75,284 (6.70)	5.85 (3.12,2.73)	2.30	2.20–2.41	<0.001
Along the sidewalk	102,560 (9.12)	4.80 (2.76,2.04)	1.88	1.80–1.96	<0.001
Mall and convenience store entrances	202,840 (18.04)	3.39 (1.96,1.43)	1.29	1.24–1.35	<0.001
Market entrances	254,400 (22.62)	3.21 (1.73,1.48)	1.23	1.18–1.28	<0.001
Public transportation (bus stops and sky train)	118,007 (10.50)	3.06 (1.54,1.52)	1.17	1.11–1.22	<0.001
Footbridges	280,067 (24.90)	3.00 (1.92,1.08)	1.15	1.10–1.19	<0.001
Inside markets	91,366 (8.14)	2.64 (1.54,1.10)	Ref		
**Date**					
Holidays	383,464 (34.10)	4.06 (2.26,1.80)	1.15	1.13–1.17	<0.001
Working days	741,060 (65.90)	3.40 (1.88,1.52)	Ref		
**Time**					
Evenings	613,625 (54.57)	3.74 (2.16,1.58)	1.15	1.13–1.18	<0.001
Mornings	510,899 (45.43)	3.27 (1.80,1.47)	Ref		
**District**					
City center	511,273 (45.47)	4.14 (2.32,1.82)	1.31	1.28–1.34	<0.001
Suburban	613,251 (54.53)	3.19 (1.83,1.36)	Ref		

### Face mask wearing divided by date and time

The proportion of people in the unprotected group was significantly lower in the morning than in the evening (3.27% and 3.74% respectively, *p*<0.001). Among the unprotected group, incorrect mask-wearing was more prevalent than non-mask-wearing both in the evening and in the morning (Table 3).

Sunday evening had the highest rate of unprotected people at 5.05%. On holidays and in the evening, people were 1.15 times more likely to be unprotected than on working days and in the morning (OR = 1.15, 95% CI 1.13–1.17, p<0.001) (Table 3).

The percentage of unprotected people during the holidays (4.06%) was higher than on working days (3.40%). Sundays and Saturdays had the highest rates of unprotected individuals at 4.98% and 3.99% respectively, while Mondays had the lowest rate at 3.23% of the 5 working days (Table 4).

**Table 4:** The distribution of unprotected group according to date and time.

Total N = 1,124,524	Monday 172,571	Tuesday 181,629	Wednesday 171,949	Thursday 165,507	Friday 164,829	Saturday 150,847	Sunday 117,192
**Morning** %Unprotected %Incorrect %Non mask	2.85 1.55 1.30	2.92 1.56 1.36	3.07 1.67 1.40	3.02 1.65 1.37	3.46 1.67 1.79	3.68 2.15 1.53	4.90 2.90 2.00
**Evening** %Unprotected %Incorrect %Non mask	3.35 1.93 1.42	3.44 1.93 1.51	3.37 1.87 1.50	3.67 2.09 1.58	3.75 2.14 1.61	4.09 2.51 1.58	5.05 2.84 2.21
**Total** %Unprotected %Incorrect %Non mask	3.23 1.82 1.41	3.30 1.81 1.49	3.35 1.87 1.48	3.47 1.95 1.52	3.55 2.00 1.55	3.99 2.39 1.60	4.98 2.90 2.08

### Face mask in different districts

**Fig 4:**
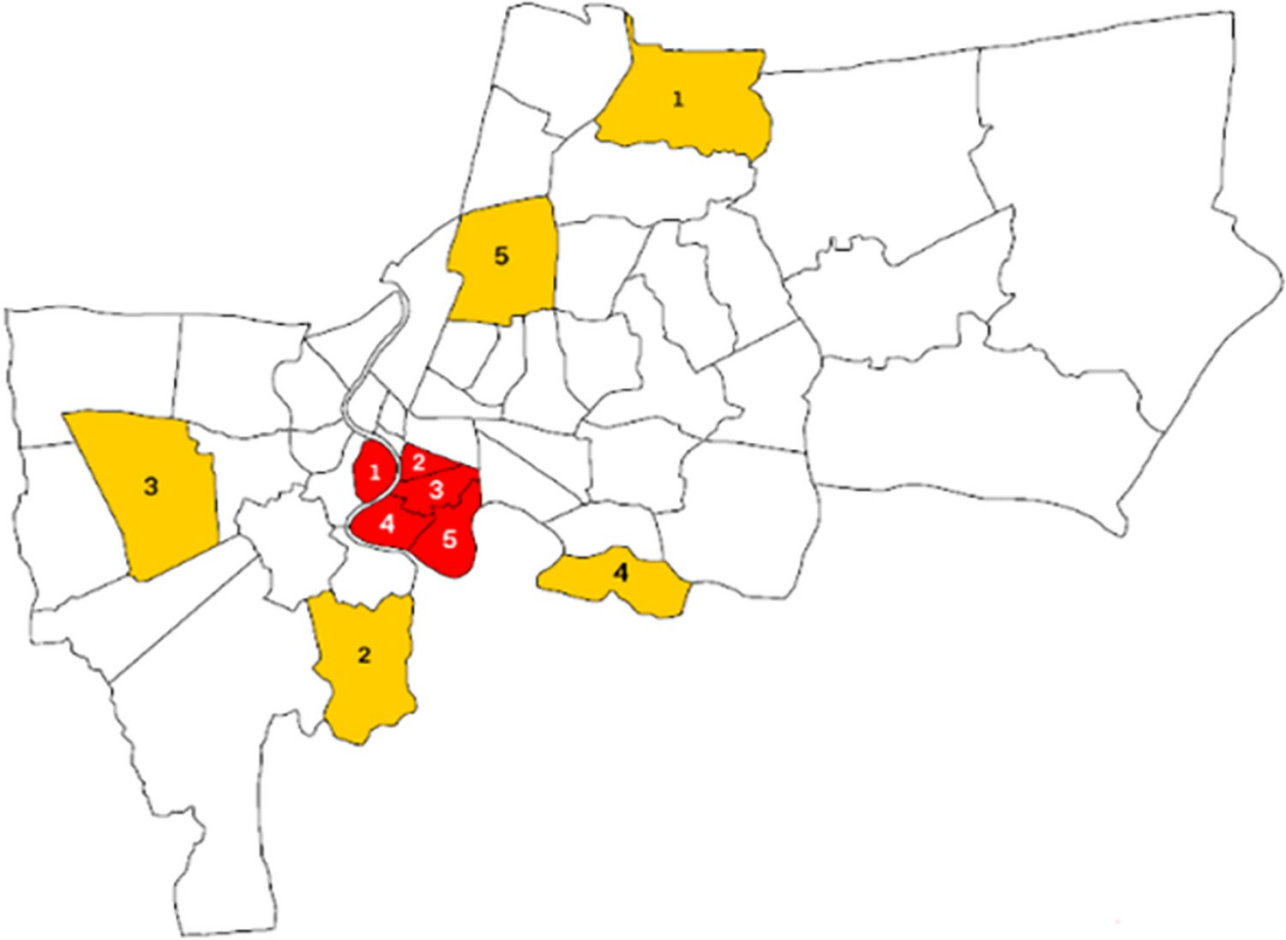
Map of Bangkok highlighting the top five districts with the highest and lowest numbers in the unprotected group. Districts with the highest unprotected group are illustrated in red: 1.Yannawa (19.4%), 2.Klong San (9.37%), 3.Sathorn (9.18%), 4.Bang Kholaem (7.62%), 5.Bang Rak (7.55%). Districts with the lowest unprotected group are illustrated in yellow: 1. Saimai (1.20%), 2. Thung Kru (1.46%), 3. Bang Khae (2.07%), 4. Bang Na (2.34%), 5. Chatuchak (2.72%).

The 5 districts with the highest proportions of unprotected people are all situated in the center of Bangkok and are adjacent to each other (Fig 4). Districts in the city center were 1.31 times more likely to have higher rates of unprotected people than suburban districts (OR = 1.31, 95% CI 1.28–1.34, *p*<0.001) (Table 3). No correlation was found between reported COVID-19 cases and unprotected people divided into each districts.

## Discussion

### AI-assisted face mask wearing (AiMASK)

Constant face mask detection is required to gather information about the public’s compliance with recommendations regarding wearing face masks. While previous studies have used manual methods to acquire this information, AiMASK-assisted face mask detection methods have allowed us to monitor a large number of people in a short period of time with high accuracy. AiMASK’s accuracy has been assessed using actual images captured from CCTVs through external validation processes. This study is the first to use AI-assisted face mask detection in real world settings. Previous studies from Egypt and China also developed a machine-learning device to detect face masks, but they only performed internal validation, with reported accuracy ranging from between 98–100% [25,26].

### Overall data

The overall rate of mask-wearing in Bangkok was 95.98%. At the beginning of the year 2021 (January 22 to February 28), the percentage of unprotected people was 2.95%, during which the number of new COVID-19 patients was at around 8.7 cases per day. Two weeks before the first cluster was announced, the proportion of unprotected individuals increased to 5.31%, reaching its maximum at 8.38%. The increase in the unprotected group was due to the lower number of new infections per day, averaging at 4.2 cases. The low number of new cases resulted in people letting their guard down.

Immediately after the first cluster was announced, the size of the unprotected group started to decline gradually. Not long after the first, the announcement of the second cluster brought about a further decrease in the size of the unprotected group, which dropped to 2.61%. When the number of patients increase rapidly, people tend to exercise more care to protect themselves.

The government has emphasized the importance of social distancing and self-protection ever since the pandemic began in Thailand in 2020. Even when the situation was improving, the public health department still encouraged everyone to keep their distance and to not drop their guard. Data provided by AiMASK showed that measures taken have not been effective enough to maintain adequate prevention. Awareness has been raised by the announcement of new outbreaks, and the longer the duration of sustained increases in new COVID-19 patients, the more the proportion of unprotected people decreases, with high correlations. This illustrates that when the public see that the situation is not showing signs of improving, they are more aware of the high risk of contracting the virus.

Interestingly, the unprotected group consisted more of incorrect mask-wearing people than of non-mask-wearing ones. The reason for improper usage of face masks might be carelessness or lack of knowledge; either way, measures should be taken to ensure not only mask usage but also correct mask usage. During the first COVID-19 outbreak in Thailand, availability of face masks was a problem, resulting in people not wearing masks; however, this was no longer a problem at the time this study was conducted.

### Global comparison

A study of the rate of mask-wearing in public in Poland, which observed 2,353 people over 3 days, found that 65–75% of people wore masks [22]. Other studies used series of photographs to estimate rates of mask wearing from 3–5 April 2020, and they found rates in Cambodia, Peru, India, Mexico, and USA of 97%, 86%, 41%, 25%, and 21% respectively [27]. Face mask wearing in France, Iran, and Hong Kong was reported at 56.4%, 45.6%, and 87% respectively [23,28,29]. A self-reported questionnaire from Brazil found 95.5% of people who wore face masks [30].

Current published studies only reported face mask use, but not it’s correctness. If worn incorrectly, the effectiveness of face masks decreases significantly, therefore it is important to use face mask accordingly.

### Face mask wearing divided by place

From our observation, the locations with the lowest unprotected rate was inside markets (2.64%). Since the start of the COVID-19 spread in Thailand, before the Bang Kae market cluster, another market cluster was reported in Samut Sakhon on December 2020. Markets became regarded as high-risk places for the SARS-COV-2 virus, leading people to believe that they had a greater chance of contracting the virus if they went to the market, causing them to take on self-protective measures such as wearing masks. In reality, although markets have a high density of people occupying a limited amount of space, conditions which lead to rapid spread, other places such as malls and closed spaces also present a high risk of virus infection.

### Face mask wearing divided by date and time

Higher rates of unprotected behavior was found during the holidays, with Sunday evening showing the highest percentage, while the lowest rates were observed on Mondays. This could be due to the desire for relaxation after a long week of working. Before the pandemic, wearing masks was not habitual, but now it is mandatory on public transportations and in the workplaces. Gradual adoption of this new habit might be the reason why on days when people are not strictly required to wear masks, they prefer not to.

A higher percentage of the unprotected group was seen in the evenings than in the mornings, showing that people have a tendency to relax protective measures more often later on in the day. Iran observed similar findings that the rate of mask wearing in the morning was significantly higher than in the evening [23].

Knowing that during holidays and evenings people are prone to be under-protected, measures should be taken to emphasize the need to maintain mask wearing throughout the day and week. Offices and schools can help encourage people to check their protection before leaving and entering their premises. Strategies to boost the economy by promoting holidays might not be the best idea during this ongoing pandemic.

### Face mask in different districts

The 5 districts containing the highest number of people in the unprotected group are all adjacent to each other and situated in central business areas. A study from France also reported the presence of independent associations between correct mask position with rural areas [28]. In contrast, the 5 districts with the highest number of COVID-19 patients were not those with the largest unprotected group. No correlation was found between reported cases and unprotected group according to districts, and this may be because the cases found in each district were reported according to where the people resided rather than where they contracted the virus.

### Strengths and limitations

This study is the first to use AI machines to detect mask wearing in public populations and had the largest number of participants in the world. We provided data over a period of 90 days which included both time frames of low contraction rates and high spread. The longest duration of data collection in previous study was over a period of 30 days [16]. We obtained data from various places across different districts and also identified those who wore masks incorrectly.

In hopes of providing a foundation for health care policies, we provided quantitative evidence of percentages of patients correctly and incorrectly wearing masks, and correlations with increased numbers of covid infections. Highlighting areas of higher rates of non-compliance could lead to new strategies aimed at decreasing the risk of incorrectly worn masks. This data is very important for policy makers, not only for COVID-19, but also for other future cases of droplet-borne respiratory tract infections.

One limitation of this study was that information was collected from public areas of a single city, Bangkok, which might not represent mask wearing in Thailand. Reported cases of COVID infection could come from home transmissions and clinical settings, which we did not gather information concerning face mask wearing inside homes and hospitals [29]. Thailand has had a large number of reported cases to come from close contact of families, and the government has released a policy encouraging people to wear masks while inside as well. Our study only addressed the public aspect of face mask wearing which may only account for some of the COVID-19 cases.

The aim of our study was not to find correlations between face mask wearing and the number of COVID-19 patients due to many reasons. One, is that we do not know when the daily reported cases were infected with the virus. Therefore, it is hard to evaluate whether patients tested positive for corona virus today were due to inadequate mask wearing last week, the week before, or even from not wearing masks in public. Secondly, wearing face mask is only one of the many alternative measures to lower rates of COVID-19 infection, it does not entirely eliminate the risk of contracting the virus.

Lastly, our current AI machine could not differentiate the different types of masks such as N95, cloth, and medical masks. Due to this new developed system, AiMASK still has its limits in grading poor quality images or images that it is not confident with. These images will then be classified into ungradable, where data would be manually allocated into the correct group or discarded later.

## Conclusion

AI-assisted face mask detection illustrates the current rates of mask wearing which we believe reflects the public’s awareness. Higher tendencies toward no protection were found in the evenings, during holidays, and in city centers. Though the overall rate of mask wearing in Bangkok is relatively high (95.98%), data has shown that lower rates of COVID-19 has led to increased numbers in the unprotected group, and whether this is a large or small percentage, it is still unprotected and presents a higher possibility of transmission, eventually leading to recurrent outbreaks. This study shows current gaps in the public’s behavior which can be adjusted to heighten self-protective measures. Policies focusing on current shortfalls will help maintain a high rate of protection. AiMASK has been developed to use images attained from CCTVs already available throughout the city, meaning that it can be used on a nationwide scale or even worldwide with high percentages of accuracy.

## Supporting information

S1 Fig Sample photos of protected and unprotected group used for internal and external validation. (TIF)

## Abbreviations

AiMASK: Artificial intelligence-assisted face mask detection
COVID-19: Corona virus disease 2019
SARS-CoV-2: Severe acute respiratory syndrome coronavirus 2

## References

[1] Liu YC, Kuo RL, Shih SR. COVID-19: The first documented coronavirus pandemic in history. Biomed J. 2020 Aug;43(4):328–333. doi: 10.1016/j.bj.2020.04.007 32387617 PMC7199674

[2] Kumar A, Singh R, Kaur J, Pandey S, Sharma V, Thakur L, et al. Wuhan to World: The COVID-19 Pandemic. Front Cell Infect Microbiol. 2021;11:596201. doi: 10.3389/fcimb.2021.596201 33859951 PMC8042280

[3] WHO 2022. Coronavirus Disease (COVID-19) dashboard. https://covid19.who.int. (accessed 16 August 2022).

[4] Mulu GB, Kebede WM, Worku SA, Mittiku YM, Ayelign B. Preparedness and Responses of Healthcare Providers to Combat the Spread of COVID-19 Among North Shewa Zone Hospitals, Amhara, Ethiopia, 2020. Infect Drug Resist. 2020 Sep 16;13:3171–3178. doi: 10.2147/IDR.S265829 33061469 PMC7520113

[5] Nicola M, Alsafi Z, Sohrabi C, Kerwan A, Al-Jabir A, Iosifidis C, et al. The socio-economic implications of the coronavirus pandemic (COVID-19): A review. Int J Surg 2020 Jun;78:185–193. doi: 10.1016/j.ijsu.2020.04.018 32305533 PMC7162753

[6] Rasheed R, Rizwan A, Javed H, Sharif F, Zaidi A. Socio-economic and environmental impacts of COVID-19 pandemic in Pakistan-an integrated analysis. Environ Sci Pollut Res Int. 2021;28(16):19926–43. doi: 10.1007/s11356-020-12070-7 33410007 PMC7787403

[7] Li Y, Mutchler JE. Older Adults and the Economic Impact of the COVID-19 Pandemic. J Aging Soc Policy. 2020;32(4–5):477–87. doi: 10.1080/08959420.2020.1773191 32543304

[8] Chen PZ, Bobrovitz N, Premji Z, Koopmans M, Fisman DN, Gu FX. Heterogeneity in transmissibility and shedding SARS-CoV-2 via droplets and aerosols. Elife. 2021 Apr 16;10:e65774. doi: 10.7554/eLife.65774 33861198 PMC8139838

[9] Jarvis MC. Aerosol Transmission of SARS-CoV-2: Physical Principles and Implications. Front Public Health. 2020 Nov 23;8:590041. doi: 10.3389/fpubh.2020.590041 33330334 PMC7719704

[10] Tabatabaeizadeh SA. Airborne transmission of COVID-19 and the role of face mask to prevent it: a systematic review and meta-analysis. Eur J Med Res. 2021;26(1):1. doi: 10.1186/s40001-020-00475-6 33388089 PMC7776300

[11] Jayaweera M, Perera H, Gunawardana B, Manatunge J. Transmission of COVID-19 virus by droplets and aerosols: A critical review on the unresolved dichotomy. Environ Res. 2020;188:109819. doi: 10.1016/j.envres.2020.109819 32569870 PMC7293495

[12] Chu DK, Akl EA, Duda S, Solo K, Yaacoub S, Schünemann HJ. Physical distancing, face masks, and eye protection to prevent person-to-person transmission of SARS-CoV-2 and COVID-19: a systematic review and meta-analysis. Lancet. 2020 Jun 27;395(10242):1973–1987. doi: 10.1016/S0140-6736(20)31142-9 32497510 PMC7263814

[13] Wiersinga WJ, Rhodes A, Cheng AC, Peacock SJ, Prescott HC. Pathophysiology, Transmission, Diagnosis, and Treatment of Coronavirus Disease 2019 (COVID-19): A Review. JAMA. 2020 Aug 25;324(8):782–793. doi: 10.1001/jama.2020.12839 32648899

[14] Bundgaard H, Bundgaard JS, Raaschou-Pedersen DET, von Buchwald C, Todsen T, Norsk JB, et al. Effectiveness of Adding a Mask Recommendation to Other Public Health Measures to Prevent SARS-CoV-2 Infection in Danish Mask Wearers: A Randomized Controlled Trial. Ann Intern Med. 2021;174(3):335–43. doi: 10.7326/M20-6817 33205991 PMC7707213

[15] Chaabna K, Doraiswamy S, Mamtani R, Cheema S. Facemask use in community settings to prevent respiratory infection transmission: A rapid review and meta-analysis. Int J Infect Dis. 2021 Mar;104:198–206. doi: 10.1016/j.ijid.2020.09.1434 32987183 PMC7518963

[16] Esposito S, Principi N, Leung CC, Migliori GB. Universal use of face masks for success against COVID-19: evidence and implications for prevention policies. Eur Respir J. 2020 Jun 18;55(6):2001260. doi: 10.1183/13993003.01260-2020 32350103 PMC7191114

[17] Leffler CT, Ing E, Lykins JD, Hogan MC, McKeown CA, Grzybowski A. Association of Country-wide Coronavirus Mortality with Demographics, Testing, Lockdowns, and Public Wearing of Masks. Am J Trop Med Hyg. 2020 Dec;103(6):2400–2411. doi: 10.4269/ajtmh.20-1015 33124541 PMC7695060

[18] Worby CJ, Chang HH. Face mask use in the general population and optimal resource allocation during the COVID-19 pandemic. Nat Commun. 2020 Aug 13;11(1):4049. doi: 10.1038/s41467-020-17922-x 32792562 PMC7426871

[19] WHO 2022. Mask use in the context of COVID-19. https://covid19.who.int. (accessed: 16 August 2022).

[20] Howard J, Huang A, Li Z, Tufekci Z, Zdimal V, van der Westhuizen HM, et al. An evidence review of face masks against COVID-19. Proc Natl Acad Sci U S A. 2021 Jan 26;118(4):e2014564118. doi: 10.1073/pnas.2014564118 33431650 PMC7848583

[21] Matuschek C, Moll F, Fangerau H, Fischer JC, Zänker K, van Griensven M, et al. Face masks: benefits and risks during the COVID-19 crisis. Eur J Med Res. 2020 Aug 12;25(1):32. doi: 10.1186/s40001-020-00430-5 32787926 PMC7422455

[22] Ganczak M, Pasek O, Duda-Duma Ł, Świstara D, Korzeń M. Use of masks in public places in Poland during SARS-Cov-2 epidemic: a covert observational study. BMC Public Health. 2021 Feb 23;21(1):393. doi: 10.1186/s12889-021-10418-3 33622279 PMC7901005

[23] Rahimi Z, Shirali GA, Araban M, Mohammadi MJ, Cheraghian B. Mask use among pedestrians during the Covid-19 pandemic in Southwest Iran: an observational study on 10,440 people. BMC Public Health. 2021 Jan 14;21(1):133. doi: 10.1186/s12889-020-10152-2 33446172 PMC7807226

[24] Chaimayo C, Kaewnaphan B, Tanlieng N, Athipanyasilp N, Sirijatuphat R, Chayakulkeeree M, et al. Rapid SARS-CoV-2 antigen detection assay in comparison with real-time RT-PCR assay for laboratory diagnosis of COVID-19 in Thailand. Virol J. 2020 Nov 13;17(1):177. doi: 10.1186/s12985-020-01452-5 33187528 PMC7665091

[25] Loey M, Manogaran G, Taha MHN, Khalifa NEM. A hybrid deep transfer learning model with machine learning methods for face mask detection in the era of the COVID-19 pandemic. Measurement (Lond). 2021 Jan 1;167:108288. doi: 10.1016/j.measurement.2020.108288 32834324 PMC7386450

[26] Qin B, Li D. Identifying Facemask-Wearing Condition Using Image Super-Resolution with Classification Network to Prevent COVID-19. Sensors (Basel). 2020 Sep 14;20(18):5236. doi: 10.3390/s20185236 32937867 PMC7570494

[27] Elachola H, Gozzer E, Rahman NMM, Ditekemena J, Pando-Robles V, Pa K, et al. Photo-epidemiology to estimate face covering use in select areas in Asia versus the Americas and Africa during the COVID-19 pandemic. J Travel Med. 2020 Dec 23;27(8):taaa121. doi: 10.1093/jtm/taaa121 32761134 PMC7454811

[28] Deschanvres C, Haudebourg T, Peiffer-Smadja N, Blanckaert K, Boutoille D, Lucet JC, et al. How do the general population behave with facemasks to prevent COVID-19 in the community? A multi-site observational study. Antimicrob Resist Infect Control. 2021 Mar 29;10(1):61. doi: 10.1186/s13756-021-00927-6 33781341 PMC8006136

[29] Tam VC, Tam SY, Poon WK, Law HKW, Lee SW. A reality check on the use of face masks during the COVID-19 outbreak in Hong Kong. EClinicalMedicine. 2020 Apr 24;22:100356. doi: 10.1016/j.eclinm.2020.100356 32337502 PMC7180351

[30] Pereira-Ávila FMV, Lam SC, Góes FGB, Gir E, Pereira-Caldeira NMV, Teles SA, et al. Factors associated with the use and reuse of face masks among Brazilian individuals during the COVID-19 pandemic. Rev Lat Am Enfermagem. 2020 Sep 7;28:e3360. doi: 10.1590/1518-8345.4604.3360 32901772 PMC7478877

