# Supplementary figures and images for: Investigating public behavior with artificial intelligence-assisted detection of face mask wearing during the COVID-19 pandemic
Source: PLoS One. 2023 Apr 11;18(4):e0281841. doi: 10.1371/journal.pone.0281841 (PMC10089330; doi:10.1371/journal.pone.0281841)

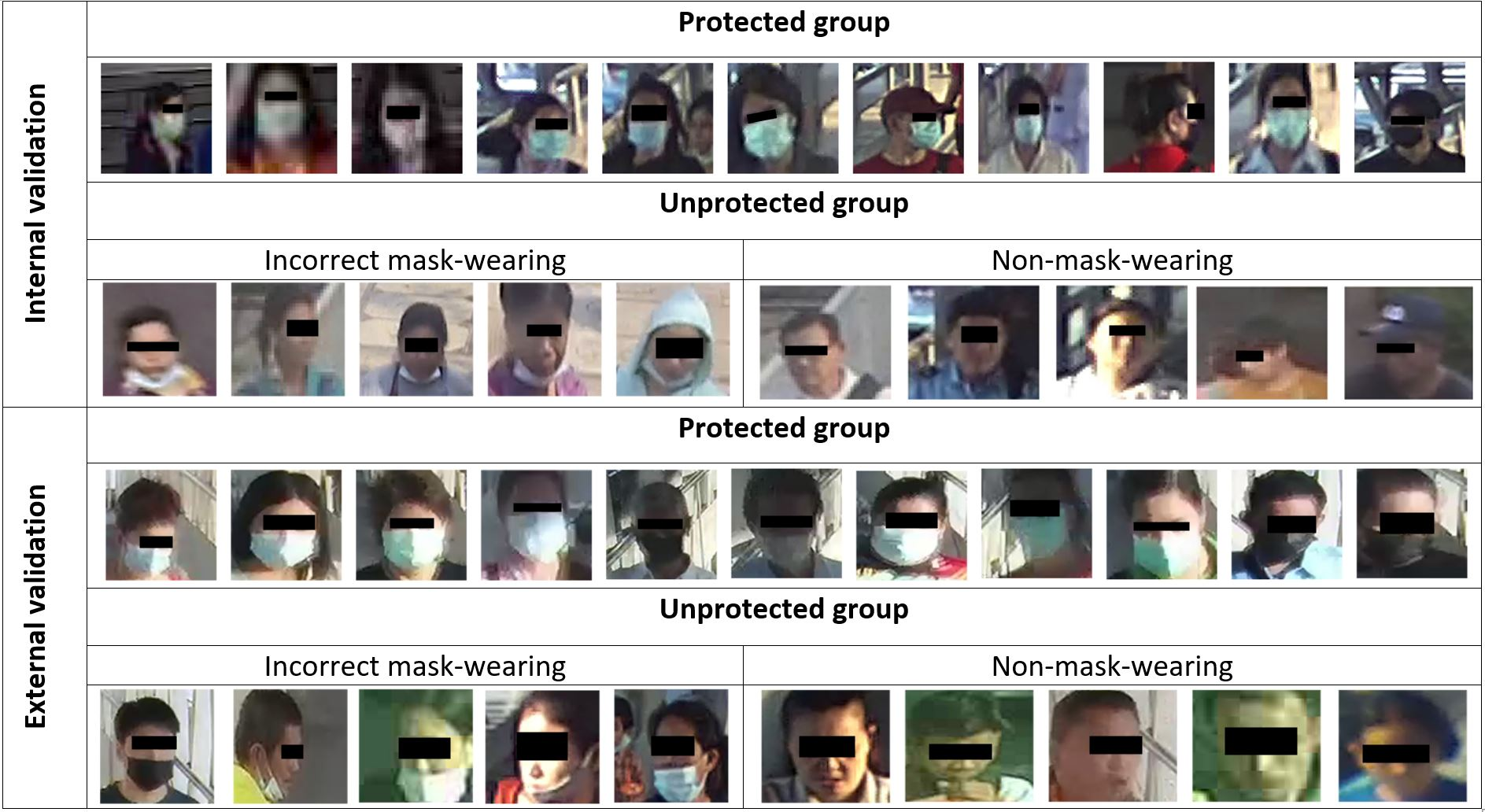

Supplement: S1 Fig — (TIF) [file pone.0281841.s001.tif]
